# Treatment options for second-stage gambiense human African trypanosomiasis

**DOI:** 10.1586/14787210.2014.959496

**Published:** 2014-09-10

**Authors:** Gilles Eperon, Manica Balasegaram, Julien Potet, Charles Mowbray, Olaf Valverde, François Chappuis

**Affiliations:** ^a^ Geneva University Hospitals, Geneva, Switzerland

**Keywords:** eflornithine, fexinidazole, gambiense, human African trypanosomiasis, melarsoprol, nifurtimox, oxaborole, SCYX-7158, second stage, sleeping sickness

## Abstract

Treatment of second-stage gambiense human African trypanosomiasis relied on toxic arsenic-based derivatives for over 50 years. The availability and subsequent use of eflornithine, initially in monotherapy and more recently in combination with nifurtimox (NECT), has drastically improved the prognosis of treated patients. However, NECT logistic and nursing requirements remain obstacles to its deployment and use in peripheral health structures in rural sub-Saharan Africa. Two oral compounds, fexinidazole and SCYX-7158, are currently in clinical development. The main scope of this article is to discuss the potential impact of new oral therapies to improve diagnosis-treatment algorithms and patients’ access to treatment, and to contribute to reach the objectives of the recently launched gambiense human African trypanosomiasis elimination program.

Human African trypanosomiasis (HAT) or sleeping sickness is a neglected tropical disease (NTD) caused by *Trypanosoma brucei*, a protozoan parasite transmitted to humans through the bite of a blood-sucking tsetse fly of the genus of *Glossina.* Two morphologically indistinguishable subspecies are responsible for the disease. These subspecies differ by their clinical presentation, epidemiology and vector species: *T. brucei gambiense*, found in western and central Africa, is responsible for a chronic form of the disease and humans are the main or sole reservoir, while *T. brucei rhodesiense* is a zoonosis found in eastern and southern Africa and generally associated with a more acute clinical presentation. In recent years, *T. brucei gambiense* has caused >95% of reported HAT cases [1,2].

The control of *T. brucei gambiense* HAT is based on mass population screening, followed by treatment of all infected patients, and vector control. In the early 1960s, transmission of the parasite was essentially interrupted in endemic areas following extensive control activities established by the colonial authorities [3]. However, neglect and political troubles led to a re-emergence of HAT that started in the mid-1970s [4]. In response to the dramatic recrudescence that peaked during the 1990s, WHO, national control programs and non-governmental organizations massively scaled up control activities. Consequently, the incidence of reported cases showed a 72% reduction between 2000 and 2011, when less than 7000 new cases were reported [5]. In 2009, only the Central African Republic (CAR) and the Democratic Republic of Congo (DRC) reported more than 1000 new cases per year. However, the true number of cases may be underestimated by a factor of 3 [3,6].

The disease occurs in two sequential stages: the first (hemato-lymphatic) stage, and the second (meningo-encephalitic) stage caused by the invasion of trypanosomes into the CNS. While the incubation period is short (<3 weeks) in rhodesiense HAT, it is longer – although not known with precision – in gambiense HAT. First-stage illness is associated with non-specific clinical features such as fever, pruritus, arthralgia, enlarged lymph nodes, fatigue and headaches, while second stage is associated with various and progressive neuropsychiatric symptoms and signs that ultimately lead to coma and death [7–9].

Because of its non-specific clinical presentation, the diagnosis of HAT requires laboratory confirmation. The diagnosis of gambiense HAT follows a three-step pathway: screening, parasitic confirmation and staging. The card agglutination test for trypanosomiasis (CATT) is widely used in the field for screening [10]. Patients with a positive CATT on whole blood or, preferably, diluted serum, undergo a microscopic search for trypanosomes in lymph node aspirate and in blood. As trypanosomes circulating in blood are usually scant, concentration methods must be used, such as the microhematocrit centrifugation technique, or the mini-anion-exchange centrifugation technique [7]. As most clinical features of the two stages overlap or are mild/absent, distinguishing the two stages on the basis of clinical features is not possible. Therefore, analysis of the cerebrospinal fluid obtained by lumbar puncture is routinely done to define the stage of the disease. Patients with trypanosomes and/or an increased (>5 cells/μl) white cell count in the cerebrospinal fluid are in second stage, while patients with no trypanosomes and ≤5 cells/μl are in first stage. Because of the complexity and various toxicity profiles of anti-trypanosomal drugs (as described below), parasitological confirmation and lumbar puncture remain routinely performed to confirm the presence of HAT and stage the illness.

HAT is usually fatal in the absence of treatment, even though cases of asymptomatic chronic infections have been described in West Africa [11]. Treatment of first-stage gambiense HAT (7–10 daily injections of pentamidine) has remained the same for over 50 years. In contrast, the treatment of second-stage gambiense HAT has drastically evolved during the last 15 years and new drugs are in the pipeline. This paper therefore focuses on past, present and future therapies for second-stage gambiense HAT and discusses how the development of new drugs will potentially impact on individual patients, and on overall disease control.

## Anti-trypanosomal drugs for second-stage gambiense HAT: a long history of a short list

In the early 20th century, the first arsenical drug (atoxyl) was developed and used against HAT. It was initially only effective on first-stage gambiense HAT. Since the discovery of tryparsamide in the 1920s, the second stage was shown to be curable. Because of the high toxicity of tryparsamide, new arsenical drugs were developed during the 1940s. One of these compounds, melarsoprol, was shown to be effective on both *T. brucei* subspecies and on both disease stages [12–14]. Since side effects were life threatening and less toxic alternatives existed for first-stage HAT, melarsoprol use has been restricted to second-stage patients. It was widely used since the 1950s and was the only available treatment for half a century until eflornithine became accessible [15]. The mode of action of melarsoprol remains unknown [16]. Various schedules have been proposed for treatment of second-stage gambiense HAT. The conventional long schedules of 2–3 series of 3–4 daily intravenous injections, separated by 1-week drug-free intervals were used for several decades. Dosages were either incremental or directly to the maximal daily dose of 3.6 mg/kg. The incremental dosage was shown to be associated with a higher relapse rate [17]. A shortened abridged schedule (2.2 mg/kg/day for 10 consecutive days) was later developed and was shown to have comparable efficacy and safety profiles to the conventional schedule. As the abridged schedule is more practical and almost halves hospitalization time [18,19], it gradually replaced conventional schedules in the early 2000s. However, increased treatment failure rates (up to 30%) due to suspected resistance have been reported in several endemic foci in the last two decades [18,20–23]. Adverse events (AE) attributable to melarsoprol are frequent and can be life-threatening. The most feared AE is an encephalopathic syndrome [24] characterized by coma and repeated convulsions, which occurs in 5–10% of patients and is fatal in around half of them [18,25,26]. The pathogenesis of encephalopathic syndrome is not fully understood but is likely to be immune-mediated.

In the mid-1980s, initial field use in Sudan showed that eflornithine (d,l,α-difluoromethylornithine) could be a potential effective therapeutic alternative for second-stage gambiense [27]. Eflornithine was initially developed for its antitumor effects, but showed antiprotozoal activity *in vitro* [28]. It is trypanostatic rather than trypanocidal. Eflornithine selectively and irreversibly inhibits the trypanosomal ornithine decarboxylase (ODC), an important enzyme in the synthesis of polyamines, which are essential for the growth and multiplication of all eukaryotic cells. Trypanosomes are more susceptible to this drug than human cells, possibly because of the slow turnover of ODC in *T. brucei gambiense.* The rapid turnover of ODC or other mechanisms, such as a higher putrescine uptake rates or decrease of *S*-adenosyl methionine metabolism, is likely to be responsible for the innate resistance of *T. brucei rhodesiense* [29,30]. Eflornithine is administered intravenously by four daily 2-h infusions of 100 mg/kg for 14 days [15]. Cure rates with this regimen is approximately 90% [22,31–34]. A 7-day regimen was shown to be less effective [35]. When administered orally, eflornithine has a bioavailability of around 50% and is associated with increased rates of diarrhea and treatment failure; it is therefore no longer used orally. AEs are frequent and similar to other cytostatic drugs, but eflornithine is overall safer than melarsoprol with reported case-fatality rates below 2%. The main AEs are temporary bone marrow suppression (anemia, leucopenia and/or thrombocytopenia), gastrointestinal symptoms and, more rarely, seizures (Table 1) [22,31–36]. Eflornithine as monotherapy was introduced as first-line treatment in field projects run by Médecins Sans Frontières (MSF) in 2000, but had a low overall uptake until 2006 when WHO launched a kit format and coordinated training of staff from national sleeping sickness control programs. In 2008, 51% of second-stage gambiense HAT patients in Africa were still treated with melarsoprol [2]. This slow development was due to the heavy logistics and human (i.e., nurses) resources needed to transport and administer the 14-day regimen in remote healthcare facilities.

**Table 1.** **Summary of published data on effectiveness and safety of drugs for second-stage gambiense human African trypanosomiasis before the nifurtimox-eflornithine combination therapy era.**

**Compound**	**Population size**	**Cure rate (%)^†^**	**In-hospital case fatality rate (%)^‡^**	**Most frequent adverse events**		**Ref.**
Melarsoprol (before 1999)	880–2221	89.7–97.2	2.2–6.5	≥1 Adverse events Encephalopathic syndrome^§^ Melarsoprol reaction^¶^ Headache Tremor Peripheral neuropathy Diarrhea Skin rash	30–75% 2.2–8.2% 4.4–32% 3.4–41.8% 17.7–20.4% 2.5–10.8% 2.2–11.7% 6.5–7.5%	[13,14]
Melarsoprol^#^ (after 1999)	62–2270	62.9–92				[18,20–25]
Eflornithine^††^	144–1055	84–95.6	0.4–3.1	Headache Seizures Anemia Leucopenia Infection Diarrhea Reaction at the injection site	31.2–45.8% 3.2–9% 41.7% 52.4% 15–17.4% 11.8–35.6% 3.5–24.6%	[22,31–35]
Nifurtimox	4–70	44–80	0–6.3	Headache Seizures Dizziness Tremor Anorexia Nausea Diarrhea Skin rash	15.7–27.3% 2.9–13.3% 10–18.2% 8–16.7% 10–40% 4–4.3% 14.3% 1.4–25%	[23,38–42]
Nifurtimoxeflornithine combination therapy	17–1735	93.5–98.4	0–1.6	≥1 Adverse events Headache Seizures Dizziness Tremor Psychiatric disorders^‡‡^ Anorexia Nausea/vomiting Diarrhea	60.1–95.1% 0–38.5% 1.4–23.5% 1.7–32.3% 0–41.9% 2.5–23.1% 4.1–25.2% 5.9–58% 1.1–23.5%	[34,44,45,48–50]

^†^To allow for comparison between studies, patients lost to follow-up were considered as cured, and patients with non-response, relapse and death (in-hospital or after discharge) were considered as treatment failures. All patients included in the studies for treatment were used as denominator. ^‡^Deaths occurring ≤30 days after treatment initiation. ^§^Defined as repeated or prolonged seizures and/or rapid deterioration of level of consciousness or coma. Deaths presumably due to encephalopathic syndromes were included, except in Bisser *et al.* [23]. ^¶^The definition used to define ‘melarsoprol reaction’ varied among studies; it refers to several symptoms and signs (e.g., headaches, tremor, hypertension) that are considered as warning signs preceding the encephalopathic syndrome. ^#^Both the conventional and abridged schedules were included. ^††^Only studies reporting the use of eflornithine administered intravenous for 14 days were included. ^‡‡^The most frequently reported psychiatric disorders were anxiety, insomnia, confusion and hallucinations.

Nifurtimox is an orally bioavailable 5-nitrofuran that has been used since the 1970s for the treatment of Chagas disease (American trypanosomiasis). Its mode of action was poorly understood until recently. As a prodrug, nifurtimox acts through being activated by an unusual nitroreductase in *T. brucei* forming a reactive nitrile, which is toxic to trypanosomes [37]. Initial case series of patients treated with various dosages and treatment durations of nifurtimox for primary or relapse gambiense HAT showed limited efficacy [23,38–42]. AEs (e.g., anorexia, nausea, neuropsychiatric disturbances) were frequent and appeared dose- and duration-dependent.

The effectiveness and safety profiles of melarsoprol, eflornithine and nifurtimox in monotherapies are summarized in Table 1.

## Development of combination treatments & entry into the nifurtimox-eflornithine combination therapy era

The rise of treatment failure rates with melarsoprol, the difficulty of scaling-up the use of the logistic-demanding standard eflornithine monotherapy and the paucity of available drugs – and the resulting need to protect against parasite resistance – triggered clinical research on combination therapies [43]. Bisser *et al.* demonstrated the efficacy of combining melarsoprol and nifurtimox but at the cost of high toxicity [23]. A study conducted in Uganda compared three different combination therapies: melarsoprol-nifurtimox (M + N), melarsoprol-eflornithine (M + E) and nifurtimox-eflornithine (N + E). The N + E combination showed clear superiority over the other two regimens in terms of both efficacy and safety. The trial was stopped for ethical reasons because of the high fatality rate observed in the M + N arm [44], but a subsequent case series of patients treated with N + E confirmed its high efficacy and safety profile [45]. In these two studies, eflornithine was administered intravenously four-times a day for 7 days.

In order to further simplify the administration of eflornithine, evaluating a twice-a-day regimen was considered a priority. The rationale of this approach was based on the argument that the short eflornithine half-life, estimated to be 3 h in plasma [46], is balanced by its long-lasting pharmacodynamic effect. Indeed, it requires 18–19 h for *T. brucei gambiense* to replenish their ODC after inhibition by eflornithine [29,47]. The twice-a-day eflornithine schedule was therefore evaluated in association with 10-day oral nifurtimox in an open-label, Phase III, randomized controlled trial in the Congos. This landmark study demonstrated the non-inferiority of the nifurtimox–eflornithine combination therapy – since then better known as nifurtimox-eflornithine combination therapy (NECT) – in comparison to standard eflornithine monotherapy, in terms of both efficacy and safety [34]. While most patients presented one or more AE (with an average of 3–4.5 AEs per patient) [34,48–50], NECT was associated with a decreased risk of major AE, defined as grade 3 or 4 of the Common Terminology Criteria for Adverse Events, compared with eflornithine monotherapy (14.0 vs 28.7%, respectively). This reduction was mainly explained by lower frequencies of fever, concomitant infections, neutropenia, hypertension and diarrhea. In contrast, NECT-treated patients were more likely to present some gastrointestinal disorders, that is, anorexia and nausea/vomiting [34]. A Phase IIIb study and two studies reporting and analyzing the data collected during a WHO-coordinated pharmacovigilance program confirmed the high effectiveness and safety of NECT in over 2000 patients (Table 1) [48–50].

In addition, the fourfold decrease in the number of intravenous infusions with NECT compared with standard eflornithine monotherapy (n = 14 vs 56) was a tremendous improvement in terms of ease of administration, cost and logistic constraints (smaller volume and lighter weight of treatment kits) [49,51]. This greatly facilitated the deployment of NECT in Africa, and over 95% of reported second-stage gambiense HAT patients were treated in 2013 with this regimen, which was included in the WHO’s Essential Medicines List in May 2009 and in the Pediatric Essential Medicines list in May 2013.

## Access to anti-trypanosomal drugs: a long battle

In the last century, pharmaceutical companies have neglected the production, registration and distribution of existing drugs for HAT. The problems faced during this period and the subsequent solutions found are useful lessons to prepare for the introduction and uptake of new diagnostics and treatments in the future.

It took more than a decade for eflornithine to become readily available at a global level and be gradually introduced in HAT programs. The drug was registered in 1990 by the US Federal Drug Administration for the treatment of second-stage gambiense HAT. However, its production was subsequently discontinued in 1995 by Hoechst Marion Roussel (one of the predecessors of Aventis, which later became Sanofi), leaving no other options than melarsoprol to treat patients with second-stage HAT. The search by WHO and MSF for a new company to take over production failed. This unacceptable situation reached a climax in 2000, when Bristol-Myers Squibb marketed a facial hair-removal cream containing eflornithine. This was the ultimate betrayal for people diagnosed with HAT; while eflornithine was made available for use as a cosmetic in wealthy countries, it was no longer produced as a life-saving drug for HAT patients. Advocacy efforts led by MSF – notably its newly created Access Campaign – and others, catalyzed the decision of Aventis to sign an agreement with WHO that included the donation of eflornithine, melarsoprol and pentamidine in unlimited quantities over the following 5 years [52]. A similar donation agreement for nifurtimox and suramin was signed in 2001 between WHO and Bayer. These 5-year donation agreements were renewed in 2006 and 2011.

The market size for HAT drugs is too small to consider a classic supply model based on competition between generic companies to secure sustainable, affordable supplies of the drugs. In that sense, Bayer and Sanofi’s long-term commitment to donate the drugs in unlimited quantities has been a pragmatic solution. However, the current construction of Sanofi’s donation has limitations. The value of the company’s donation of HAT drugs is subtracted from the US$5,000,000 annual grant given by Sanofi to WHO to support NTD control programs. As a result, when the volume of Sanofi’s drugs donation rises, the complementary funding received by WHO from Sanofi to support national control programs is accordingly reduced. This has occurred over the last few years with the rapid uptake of NECT and the phasing-out of melarsoprol for second-stage HAT, the former treatment being much more expensive to produce, store and transport than the latter [53]. More predictable funding of the WHO NTD program is recommended to compensate for this volatility.

WHO coordinates the demand forecast and drug orders to Bayer and Sanofi, 2 years ahead of the distribution of the drugs. As a small proportion of patients treated with NECT relapse, the demand for melarsoprol for gambiense HAT is extremely low. However, Sanofi has been committed to continuing the production of melarsoprol. In the event a new drug gets recommended as first-line treatment for second-stage gambiense HAT, it will be critical to continue the production of eflornithine and nifurtimox, as NECT will still be needed as second-line treatment.

In addition to the lack of production, there have been additional obstacles for HAT patients to access drugs in the past:

- In the early 2000s, most patients could not be treated with eflornithine though the drug itself was free-of-charge, because of a lack of resources to cover the cost of materials needed for its intravenous administration. This led WHO to design and distribute free-of-charge eflornithine (and later NECT) kits, which include all the necessary items to administer treatment, such as drugs, bags of sterile water for injection, catheters, etc. The NECT kit now contains treatment material for four patients. It weighs 39 kg and its volume is 170 dm^3^. This format, resembling that of a standard vaccines cold box, remains a logistic challenge for transportation to remote rural hospitals. MSF Logistique in Bordeaux, France, stores all required material and anti-trypanosomal drugs, prepares and ships the kits following a WHO-coordinated plan to national sleeping sickness control programs via the WHO Office in the country. Complementary additional NECT kit formats, including a pediatric kit with the newly available pediatric formulation of nifurtimox, and a single treatment kit, for countries with no recently reported cases, are also being considered (Simarro P, Pers. Comm.).
- In the late 2000s, the introduction of NECT was jeopardized by the lack of registration of nifurtimox in HAT-endemic countries. The inclusion of NECT in the WHO essential medicines list in 2009 prompted its authorization, importation and adoption in endemic countries. The new global regulatory mechanisms designed for drugs for NTDs, including Article 58 of the EMA, could be used in the future to fast-track the registration of new drugs for HAT with the contribution of experts from both European and African regulatory agencies [54].

## New drugs in the pipeline

While NECT has been adopted as first-line treatment for second-stage gambiense HAT in nearly all endemic countries, its practical limitations restrict its use to hospital settings. Simpler diagnostic and treatment tools are required to meet the WHO objective of HAT elimination, as this goal implies some degree of reintegration of HAT patients care in the existing healthcare structures.

A target product profile for a new anti-trypanosomal drug against second-stage gambiense HAT has been developed and revised regularly (Table 2). The target product profile states the need for an oral treatment, no longer than 7 days, which is safe and effective against both forms of the disease and against both stages, stable in tropical conditions and with substantially reduced cost compared with NECT.

**Table 2.** **Target product profile of a new treatment against both stage 1 and 2 gambiense human African trypanosomiasis.**

**Ideal**	**Acceptable**	**NECT (current first-line treatment)**
Effective against stage 1 and 2	Effective against stage 1 and 2 (used against stage 2 only)	Stage 1 and 2 (used against stage 2 only)
Broad spectrum (gambiense and rhodesiense)	Efficacy against gambiense only	Gambiense
Clinical efficacy >95% at 18 months follow-up	To be determined in an expert consultation	Clinical efficacy 96.5% [34]
Effective in melarsoprol refractory patients		Effective
<0.1% drug-related mortality	<1% drug possibly related mortality	1.2% possibly related mortality [49]
Safe also during pregnancy, for breast-feeding women and children		No specific adverse event found in babies born or being breast-fed after treatment [49]
Adult and pediatric formulations (rectal?)		Eflornithine pediatric dosing available + nifurtimox 120 mg tablets to be cut
No monitoring for adverse events	Weekly simple laboratory testing	Hospitalization required
<7 days p.o. once daily	10 days p.o. (up to t.i.d.)	7 days iv. infusion (b.i.d.) + 10 days p.o. (t.i.d.)
<7 days im. once daily	<10 days im. once daily	
Stability in zone 4 for >3 years	Stability in zone 4 for >12 months	Stability in zone 4 for >24 months
Trypanocidal	Trypanostatic	Trypanostatic (eflornithine) and trypanocidal (nifurtimox)
Multi-target	Unique target (but not uptake via P2-transporter only)	
<€30/course^†^ (only drug cost)	<€100^†^/course	€288^†^/course (in 4 treatments kits; WHO) [53]
	<€200^†^/course if very good on other criteria	

Definition: zone 4: hot and humid climate; temperature: 30°C, relative humidity: 60%. ^†^Includes transport to the clinical site. b.i.d.: Twice daily; im.: Intramuscular; NECT: Nifurtimox-eflornithine combination therapy; p.o.: Per os; t.i.d.: Three-times daily. Adapted from [70].

Two drugs are currently being evaluated in clinical trials for gambiense HAT:

Fexinidazole, a 5-nitroimidazole, is being evaluated in a Phase II/III randomized controlled trial sponsored by the Drugs for Neglected Diseases Initiative (DNDi) in second-stage gambiense HAT patients, in nine clinical trial centers in the DRC and CAR. Two additional cohort studies have been added recently as a ‘plug in’ to the pivotal trial. The first includes adult patients with first or early second stage, and the second includes children above 6 years of age and over 20 kg in weight with both disease stages. After being rediscovered through a careful review of existing nitroimidazole candidates, fexinidazole was shown to be effective *in vitro* and in animal models against both species of the human trypanosome. Preclinical development led to determination of its safety and the start of a Phase I clinical trial in September 2009 [55].

**Figure 1.**
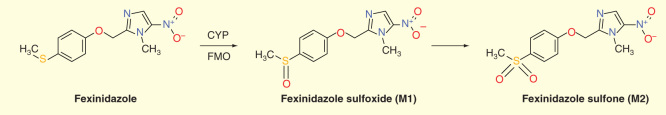
**Metabolism of fexinidazole into fexinidazole sulfoxide and sulfone by cytochrome P450 and flavin-containing monooxygenase.**

CYP: Cytochrome P450; FMO: Flavin-containing monooxygenase.

Fexinidazole acts as a prodrug and is rapidly metabolized *in vivo –* via two different pathways, cytochrome P450 and flavin-containing monooxygenase – into two metabolites: sulfoxide and sulfone (Figure 1). The therapeutic dose was determined in human volunteers, ethnicity-matched to patients in sub-Saharan Africa including a population pharmacokinetic simulation. The most active metabolite is the sulfone, which has a plasma half-life in humans of 24 h, thus allowing for a single daily dosage. An important food effect led to administration during 10 days with food, including a loading dose during the initial 4 days [56].

**Figure 2.**
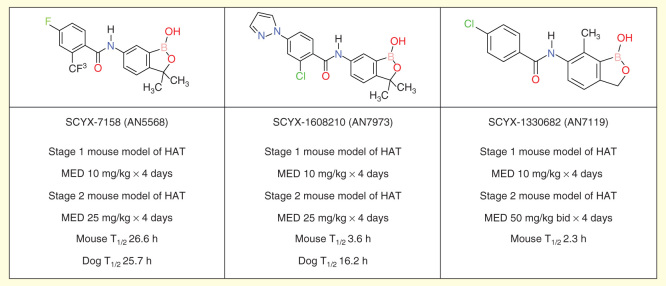
**Preclinical profiles of SCYX-7158 and two potential back-up compounds.**

The second drug in clinical trials is SCYX-7158 (AN5568), an orally available benzoxaborole (Figure 2). Successful preclinical studies have brought this compound into Phase I clinical development. The preliminary data of this ongoing, ascending dose study are consistent with a single oral dose treatment [57].

MED: Minimum effective dose; T_1/2_: Half-life.

DNDi and partners have endeavored to further optimize both the nitroimidazole and benzoxaborole series to provide further development candidates as improvements beyond fexinidazole and SCYX-7158.

**Figure 3.**
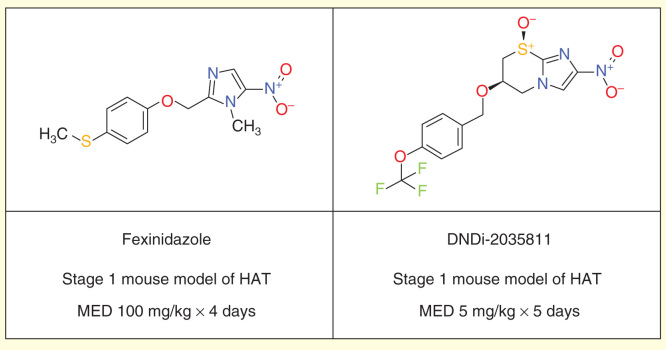
**Fexinidazole and DNDi-2035811.**

Although fexinidazole shows only moderate *in vitro* activity against *T. brucei*, it has shown great promise due to good *in vivo* activity in both first- and second-stage mouse models of HAT [55], despite having perceived risks due to the relatively high predicted human dose, limited solubility and food effect on oral bioavailability. Thus, other nitroimidazoles were sought with improved *in vitro* potency while retaining good pharmacokinetic properties and blood–brain barrier penetration, to improve still further on fexinidazole and other leads such as RJ-164 [58]. The leading compound resulting from these efforts is DNDi-2035811, identified through a partnership with the Global Alliance for TB Drug Development and the University of Auckland (Figure 3).

MED: Minimum effective dose.

The preclinical pharmacokinetic profile of SCYX-7158 identified the possibility that the compound might have a long elimination half-life in humans; a possibility that was confirmed in early Phase I trials. In case the development of this exceptional drug should run into unforeseen problems, a program was launched to identify back-up compounds with shorter predicted half-lives in human. Two advanced leads, SCYX-1608210 (AN7973) and SCYX-1330682 (AN7119), were prioritized for further study based on detailed pharmacokinetic analysis and are predicted to have shorter human half-lives (Figure 2).

**Figure 4.**
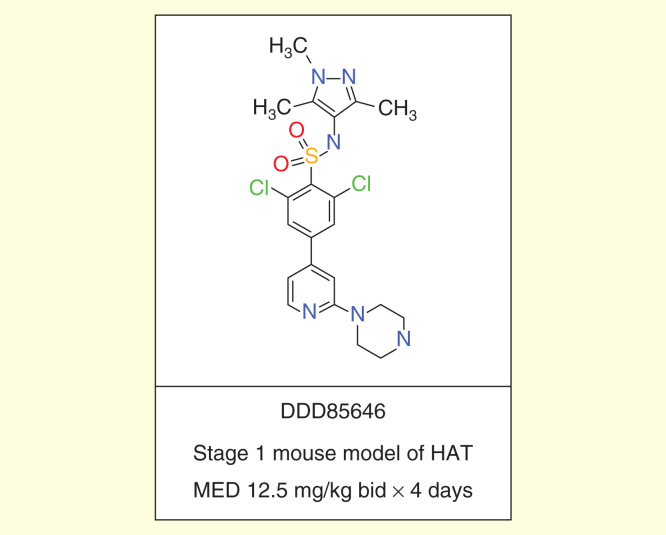
**DDD85646.**

Numerous groups continue to explore new target-based and phenotypic screening-based approaches to deliver new development candidates for treating HAT. The Dundee Drug Discovery Unit has made significant progress in the development of selective inhibitors of *T. brucei* *N*-myristoyl transferase, which show excellent activity against *T. brucei* in culture and in a stage 1 mouse model of HAT (Figure 4) [59,60]. Further optimization of these leads is needed to give improved blood–brain barrier penetration and activity in a stage 2 model of HAT.

MED: Minimum effective dose.

**Figure 5.**
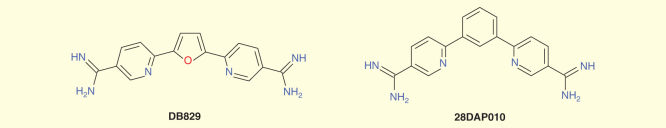
**Diamidines.**

Significant efforts have been made to develop an orally active diamidine drug for stage 2 HAT building on the earlier prototypes furamidine (DB75) and its orally active prodrug pafuramidine maleate (DB289). Although the newest members of this class such as DB829 and 28DAP010 show efficacy in a model of stage 2 HAT by parenteral administration, they are still not fully effective when administered by the oral route (Figure 5) [61,62].

Last year, teams at GlaxoSmithKline and Novartis have made significant investments in early drug discovery for HAT, moves which should help ensure a continued pipeline of quality new candidates with the potential to treat HAT [63,64].

## Expert commentary & five-year view

The progress made in the treatment and overall control of gambiense HAT over the last two decades in undeniable. Numerous lives have been saved by switching from the arsenic-based melarsoprol to eflornithine monotherapy and later NECT, which is safe and highly efficacious, for treatment of second-stage gambiense HAT. The decreasing number of reported cases of gambiense HAT over the last decade fostered the decision of WHO to launch a global HAT elimination plan with the objective of reaching <0.01% prevalence of gambiense HAT in 90% of existing endemic foci, with <2000 reported cases annually by the year 2020 [5].

The defined strategy for eliminating gambiense HAT relies on three pillars: active case detection by mobile teams, passive case detection in sentinel sites and vector control [8]. Passive case detection will reliably take place at primary (e.g., healthcare center) and secondary (e.g., district hospital) level, which implies some degree of integration of diagnostic and treatment activities within the existing public health structures. This integration is challenging because of the lack of trained staff and logistic resources in public health structures located in remote areas of most HAT affected countries (e.g., DRC, CAR) and the current complexity of diagnostic algorithms and treatment of second-stage illness (NECT).

It is therefore crucial to drastically simplify the current diagnosis and treatment approach to facilitate its implementation. The recent development and commercialization of individual serological rapid diagnostic tests (RDT) is a first step in this direction [65,66]. Their diagnostic performance has proved to be as good as the CATT for screening, and field implementation and evaluations have started in several endemic foci. The development of an oral, cheap, effective and safe drug is an essential second step and the current clinical trials evaluating fexinidazole and SCYX-7158 (see above) are very encouraging and unique in the history of this neglected condition.

**Figure 6.**
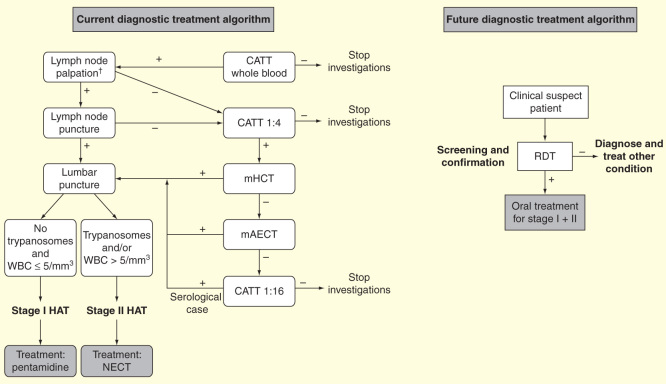
**Present and potential future diagnosis treatment algorithms for second-stage gambiense human African trypanosomiasis.**

CATT: Card agglutination trypanosoma test; HAT: Humana African trypanosomiasis; mAECT: Minianion-exchange centrifugation technique; mHCT: Micro-hematocrit centrifugation technique; NECT: Nifurtimox-eflornithine combination therapy; RDT: Rapid diagnostic test; WBC: White blood cells.

A safe and effective oral drug active on both stages of the disease would eliminate the need for staging the disease. Dropping lumbar puncture would likely improve people’s adherence to HAT detection programs as this procedure is disliked by populations [67]. An excellent safety profile of the drug may even allow elimination of the need for parasitological confirmation, as a certain tolerance of diagnostic uncertainty would then be acceptable, as is the case for RDT-based diagnosis of malaria. The elimination of disease staging and parasitological confirmation would drastically simplify the diagnostic approach and increase its feasibility and deployment in peripheral health structures. Patients would then be treated with an oral drug on-site, based on a high probability of disease, defined by the presence of suggestive clinical features (a field being re-explored) and a positive RDT result (Figure 6) [68].

The future of HAT drug development will only deliver its promise for control and elimination, if sustainable mechanisms for payment of drugs, diagnostics, supply and distribution as well as control and surveillance activities are maintained. It is essential that new drugs are considered for donation programs while old drugs are not discontinued. The format of the HAT kits will have to be adapted to new treatment tools. The development and deployment of a simpler treatment for gambiense HAT will reach its full impact only if it is closely linked with the development and deployment of simpler diagnostic tools and strategies. Therefore, a specific strategy for access to HAT diagnostics should complement the current successful drug access framework, as there is currently no global mechanism for endemic countries to purchase and distribute diagnostic tools wherever they are needed for case-finding and disease surveillance.

The recent launch of the WHO network for gambiense HAT elimination, a network that includes coordinators of HAT national programs, researchers, implementing partners and donors, will contribute to harmonize the development and deployment of new tools and strategies that will be necessary to eliminate this scourge, hopefully once for all, in sub-Saharan Africa [69].

## Notes

Key issues

- Human African trypanosomiasis (HAT) is a neglected tropical disease transmitted by tsetse flies in sub-Saharan Africa; over 95% of cases are caused by *Trypanosoma brucei gambiense*. Gambiense HAT evolves from first (hemolymphatic) to second (meningoencephalitic) stage and is generally lethal if left untreated.
- Treatment of second-stage gambiense HAT relied on melarsoprol, an arsenic-based derivative, for over 50 years. Melarsoprol is associated with a wide range of adverse effects, the most feared being an encephalopathic syndrome that kills 3–6% of patients.
- Eflornithine-based treatments, initially developed and used in monotherapy for 14 days and more recently for 7 days in combination with 10-day oral nifurtimox (NECT), are associated with improved safety and efficacy profiles; NECT has been adopted as first-line treatment for second-stage gambiense HAT in all endemic countries.
- Access to newer anti-trypanosomal drugs, in particular eflornithine, has been a long battle; donation agreements between WHO and the producers, which included funding for preparation, storage and transport of kits, drastically improved patients’ access to treatment.
- While NECT is safe and very effective, its use in remote endemic areas is impaired by the heavy nursing and logistic resources required for it to be properly administered.
- The decreasing trend in reported cases triggered WHO to launch a gambiense HAT elimination program with various partners. The strategy includes partial reintegration of control activities within existing health structures, which usually have low human and logistic resources in rural sub-Saharan Africa.
- Two oral drugs are being evaluated in clinical trials: fexinidazole, a 5-nitroimidazole, and SCYX-7158 (AN5568), a benzoxaborole. Provided that these oral drugs show good safety and efficacy profiles, they may lead to drastic simplification of diagnostic algorithms and better patient access to treatment in peripheral health structures.
- The development of simplified diagnosis-treatment algorithms, incorporating the newly developed rapid diagnostic tests and oral therapies, will also facilitate passive case detection and disease surveillance, which are crucial requirements to reach sustained disease elimination.

## References

[CIT0001] Burri C, Chappuis F, Brun R, Farrar J, Hotez PJ, Junghanss T, Gagandeep K, Lalloo D, White NJ (2014). Human African trypanosomiasis. Manson’s Tropical Diseases (23).

[CIT0002] World Health Organization (2010). Working to overcome the global impact of neglected diseases: first WHO report on neglected tropical diseases.

[CIT0003] Simarro PP, Diarra A, Ruiz Postigo JA (2011). The human African trypanosomiasis control and surveillance programme of the World Health Organization 2000-2009: the way forward. PLoS Negl Trop Dis.

[CIT0004] Pepin J, Meda HA (2001). The epidemiology and control of human African trypanosomiasis. Adv Parasitol.

[CIT0005] World Health Organization (2013). Sustaining the drive to overcome the global impact of neglected tropical diseases. Second WHO report on neglected tropical diseases.

[CIT0006] Tong J, Valverde O, Mahoudeau C (2011). Challenges of controlling sleeping sickness in areas of violent conflict: experience in the Democratic Republic of Congo. Confl Health.

[•] Chappuis F, Loutan L, Simarro P (2005). Options for field diagnosis of human African trypanosomiasis. Clin Microbiol Rev.

[••] World Health Organization (2013). Control and surveillance of human African trypanosomiasis. WHO Technical Report Series. (984).

[•] Blum J, Schmid C, Burri C (2006). Clinical aspects of 2541 patients with second stage human African trypanosomiasis. Acta Trop.

[CIT0010] Magnus E, Vervoort T, Van Meirvenne N (1978). A card-agglutination test with stained trypanosomes (C.A.T.T.) for the serological diagnosis of T. B. gambiense trypanosomiasis. Ann Soc Belg Med Trop.

[CIT0011] Jamonneau V, Ilboudo H, Kabore J (2012). Untreated human infections by Trypanosoma brucei gambiense are not 100% fatal. PLoS Negl Trop Dis.

[•] Ollivier G, Legros D (2001). Human African trypanosomiasis: a history of its therapies and their failures. Trop Med Int Health.

[CIT0013] Friedheim EA (1951). Mel B in the treatment of tryparsamide resistant T. gambiense sleeping sickness: observations on drug resistance in the trypanosomes of the French Cameroun. Am J Trop Med Hyg.

[CIT0014] Pepin J, Mpia B (2005). Trypanosomiasis relapse after melarsoprol therapy. Emerg Infect Dis.

[CIT0015] Pepin J, Milord F (1994). The treatment of human African trypanosomiasis. Adv Parasitol.

[CIT0016] Fairlamb AH, Carter NS, Cunningham M, Smith K (1992). Characterisation of melarsen-resistant Trypanosoma brucei with respect to cross-resistance to other drugs and trypanothione metabolism. Mol Biochem Parasitol.

[CIT0017] Pepin J, Mpia B (2006). Randomized controlled trial of three regimens of melarsoprol in the treatment of Trypanosoma brucei gambiense trypanosomiasis. Trans R Soc Trop Med Hyg.

[CIT0018] Schmid C, Richer M, Bilenge CM (2005). Effectiveness of a 10-Day melarsoprol schedule for the treatment of late-stage human african trypanosomiasis: confirmation from a Multinational Study (Impamel II). J Infect Dis.

[CIT0019] Burri C, Nkunku S, Merolle A (2000). Efficacy of new, concise schedule for melarsoprol in treatment of sleeping sickness caused by Trypanosoma brucei gambiense: a randomised trial. Lancet.

[CIT0020] Legros D, Evans S, Maiso F (1999). Risk factors for treatment failure after melarsoprol for Trypanosoma brucei gambiense trypanosomiasis in Uganda. Trans R Soc Trop Med Hyg.

[CIT0021] Stanghellini A, Josenando T (2001). The situation of sleeping sickness in Angola: a calamity. Trop Med Int Health.

[CIT0022] Balasegaram M, Harris S, Checchi F (2006). Melarsoprol versus eflornithine for treating late-stage Gambian trypanosomiasis in the Republic of the Congo. Bull World Health Organ.

[CIT0023] Bisser S, N’Siesi FX, Lejon V (2007). Equivalence trial of melarsoprol and nifurtimox monotherapy and combination therapy for the treatment of second-stage Trypanosoma brucei gambiense sleeping sickness. J Infect Dis.

[CIT0024] Blum J, Nkunku S, Burri C (2001). Clinical description of encephalopathic syndromes and risk factors for their occurrence and outcome during melarsoprol treatment of human African trypanosomiasis. Trop Med Int Health.

[CIT0025] Eperon G, Schmid C, Loutan L, Chappuis F (2007). Clinical presentation and treatment outcome of sleeping sickness in Sudanese pre-school children. Acta Trop.

[CIT0026] Seixas J (2004). Investigations on the encephalopathic syndrome during melarsoprol treatment of human African trypanosomiasis. Instituo de Higiene e medicina tropical.

[CIT0027] Van Nieuwenhove S, Schechter PJ, Declercq J (1985). Treatment of gambiense sleeping sickness in the Sudan with oral DFMO (DL-alpha-difluoromethylornithine), an inhibitor of ornithine decarboxylase; first field trial. Trans R Soc Trop Med Hyg.

[CIT0028] Bacchi CJ, Nathan HC, Hutner SH (1980). Polyamine metabolism: a potential therapeutic target in trypanosomes. Science.

[CIT0029] Iten M, Mett H, Evans A (1997). Alterations in ornithine decarboxylase characteristics account for tolerance of Trypanosoma brucei rhodesiense to D,L-alpha-difluoromethylornithine. Antimicrob Agents Chemother.

[CIT0030] Bacchi CJ, Garofalo J, Ciminelli M (1993). Resistance to DL-alpha-difluoromethylornithine by clinical isolates of Trypanosoma brucei rhodesiense. Role of S-adenosylmethionine. Biochem Pharmacol.

[CIT0031] Priotto G, Pinoges L, Fursa IB (2008). Safety and effectiveness of first line eflornithine for Trypanosoma brucei gambiense sleeping sickness in Sudan: cohort study. BMJ.

[CIT0032] Milord F, Pepin J, Loko L (1992). Efficacy and toxicity of eflornithine for treatment of Trypanosoma brucei gambiense sleeping sickness. Lancet.

[CIT0033] Chappuis F, Udayraj N, Stietenroth K (2005). Eflornithine is safer than melarsoprol for the treatment of second-stage Trypanosoma brucei gambiense human African trypanosomiasis. Clin Infect Dis.

[••] Priotto G, Kasparian S, Mutombo W (2009). Nifurtimox-eflornithine combination therapy for second-stage African Trypanosoma brucei gambiense trypanosomiasis: a multicentre, randomised, phase III, non-inferiority trial. Lancet.

[CIT0035] Pepin J, Khonde N, Maiso F (2000). Short-course eflornithine in Gambian trypanosomiasis: a multicentre randomized controlled trial. Bull World Health Organ.

[CIT0036] Burri C, Brun R (2003). Eflornithine for the treatment of human African trypanosomiasis. Parasitol Res.

[CIT0037] Hall BS, Bot C, Wilkinson SR (2011). Nifurtimox activation by trypanosomal type I nitroreductases generates cytotoxic nitrile metabolites. J Biol Chem.

[CIT0038] Janssens PG, De Muynck A (1977). Clinical trials with “nifurtimox” in African trypanosomiasis. Ann Soc Belg Med Trop.

[CIT0039] Moens F, De Wilde M, Ngato K (1984). Clinical trial of nifurtimox in human African trypanosomiasis. Ann Soc Belg Med Trop.

[CIT0040] Pepin J, Milord F, Mpia B (1989). An open clinical trial of nifurtimox for arseno-resistant Trypanosoma brucei gambiense sleeping sickness in central Zaire. Trans R Soc Trop Med Hyg.

[CIT0041] Pepin J, Milord F, Meurice F (1992). High-dose nifurtimox for arseno-resistant Trypanosoma brucei gambiense sleeping sickness: an open trial in central Zaire. Trans R Soc Trop Med Hyg.

[CIT0042] Van Nieuwenhove S, Declercq J (1981). Nifurtimox (lampit) treatment in late stage of gambiense sleeping sickness.

[CIT0043] Van Nieuwenhove S (2000). Gambiense sleeping sickness: re-emerging and soon untreatable?. Bull World Health Organ.

[CIT0044] Priotto G, Fogg C, Balasegaram M (2006). Three drug combinations for late-stage Trypanosoma brucei gambiense sleeping sickness: a randomized clinical trial in Uganda. PLoS Clin Trials.

[CIT0045] Checchi F, Piola P, Ayikoru H (2007). Nifurtimox plus Eflornithine for late-stage sleeping sickness in Uganda: a case series. PLoS Negl Trop Dis.

[CIT0046] Barrett MP, Boykin DW, Brun R, Tidwell RR (2007). Human African trypanosomiasis: pharmacological re-engagement with a neglected disease. Br J Pharmacol.

[CIT0047] Priotto G, Kasparian S, Ngouama D (2007). Nifurtimox-eflornithine combination therapy for second-stage Trypanosoma brucei gambiense sleeping sickness: a randomized clinical trial in Congo. Clin Infect Dis.

[CIT0048] Franco JR, Simarro P, Diarra A (2012). Monitoring the use of nifurtimox-eflornithine combination therapy (NECT) in the treatment of second stage gambiense human African trypanosomiasis. Res Rep Trop Med.

[CIT0049] Schmid C, Kuemmerle A, Blum J (2012). In-hospital safety in field conditions of nifurtimox eflornithine combination therapy (NECT) for T. b. gambiense sleeping sickness. PLoS Negl Trop Dis.

[CIT0050] Alirol E, Schrumpf D, Amici Heradi J (2013). Nifurtimox-eflornithine combination therapy for second-stage gambiense human African trypanosomiasis: medecins Sans Frontieres experience in the Democratic Republic of the Congo. Clin Infect Dis.

[CIT0051] Yun O, Priotto G, Tong J (2010). NECT is next: implementing the new drug combination therapy for Trypanosoma brucei gambiense sleeping sickness. PLoS Negl Trop Dis.

[CIT0052] Coyne PE (2001). The eflornithine story. J Am Acad Dermatol.

[CIT0053] Simarro PP, Franco J, Diarra A (2012). Update on field use of the available drugs for the chemotherapy of human African trypanosomiasis. Parasitology.

[CIT0054] Moran M, Strub-Wourgaft N, Guzman J (2011). Registering new drugs for low-income countries: the African challenge. PLoS Med.

[CIT0055] Torreele E, Bourdin Trunz B, Tweats D (2010). Fexinidazole–a new oral nitroimidazole drug candidate entering clinical development for the treatment of sleeping sickness. PLoS Negl Trop Dis.

[CIT0056] Tarral A, Blesson S, Mordt OV (2014). Determination of an optimal dosing regimen for fexinidazole, a novel oral drug for the treatment of human African trypanosomiasis: first-in-human studies. Clin Pharmacokinet.

[CIT0057] Jacobs RT, Nare B, Wring SA (2011). SCYX-7158, an orally-active benzoxaborole for the treatment of stage 2 human African trypanosomiasis. PLoS Negl Trop Dis.

[CIT0058] Trunz BB, Jedrysiak R, Tweats D (2011). 1-Aryl-4-nitro-1H-imidazoles, a new promising series for the treatment of human African trypanosomiasis. Eur J Med Chem.

[CIT0059] Frearson JA, Brand S, McElroy SP (2010). N-myristoyltransferase inhibitors as new leads to treat sleeping sickness. Nature.

[CIT0060] Brand S, Cleghorn LA, McElroy SP (2012). Discovery of a novel class of orally active trypanocidal N-myristoyltransferase inhibitors. J Med Chem.

[CIT0061] Wenzler T, Yang S, Braissant O (2013). Pharmacokinetics, Trypanosoma brucei gambiense efficacy, and time of drug action of DB829, a preclinical candidate for treatment of second-stage human African trypanosomiasis. Antimicrob Agents Chemother.

[CIT0062] Wenzler T, Yang S, Patrick DA (2014). In vitro and in vivo evaluation of 28dap010, a novel diamidine for treatment of second-stage African sleeping sickness. Antimicrob Agents Chemother.

[CIT0063] GlaxoSmithKline Partnerships: neglected tropical diseases. http://www.gsk.com/partnerships/neglected-tropical-diseases.html.

[CIT0064] Novartis Novartis Institute for Tropical Diseases (NITD). http://www.nibr.com/research/developing_world/NITD/index.shtml.

[CIT0065] Büscher P, Gilleman Q, Lejon V (2013). Rapid diagnostic test for sleeping sickness. N Engl J Med.

[CIT0066] Büscher P, Mertens P, Leclipteux T (2014). Sensitivity and specificity of HAT Sero-K-SeT, a rapid diagnostic test for serodiagnosis of sleeping sickness caused by Trypanosoma brucei gambiense: a case-control study. Lancet Glob Health.

[CIT0067] Robays J, Lefevre P, Lutumba P (2007). Drug toxicity and cost as barriers to community participation in HAT control in the Democratic Republic of Congo. Trop Med Int Health.

[CIT0068] Palmer JJ, Surur EI, Goch GW (2013). Syndromic algorithms for detection of gambiense human African trypanosomiasis in South Sudan. PLoS Negl Trop Dis.

[CIT0069] World Health Organization Stakeholders call on WHO to lead a network aimed at elimination of human African trypanosomiasis (2014). http://www.who.int/trypanosomiasis_african/meeting_declaration_2014_intro/en/.

[CIT0070] Target product profile of new treatments for human African trypanosomiasis. http://www.dndi.org/diseases-projects/diseases/hat/target-product-profile.html.

